# Molecular Basis of Recognition of Human Osteopontin by 23C3, a Potential Therapeutic Antibody for Treatment of Rheumatoid Arthritis

**DOI:** 10.1016/j.jmb.2008.07.075

**Published:** 2008-10-17

**Authors:** Jiamu Du, Sheng Hou, Chen Zhong, Zheng Lai, Hui Yang, Jianxin Dai, Dapeng Zhang, Hao Wang, Yajun Guo, Jianping Ding

**Affiliations:** 1 State Key Laboratory of Molecular Biology, Institute of Biochemistry and Cell Biology, Shanghai Institutes for Biological Sciences, Chinese Academy of Sciences, 320 Yue-Yang Road, Shanghai 200031, China; 2 Graduate School of Chinese Academy of Sciences, 320 Yue-Yang Road, Shanghai 200031, China; 3 International Joint Cancer Institute, Second Military Medical University, 800 Xiang-Yin Road, Shanghai 200433, China

**Keywords:** mAb, monoclonal antibody, RA, rheumatoid arthritis, SF, synovial fluid, CIA, collagen-induced arthritis, CDR, complementarity-determining region, BSA, buried surface area, FWR, framework region, osteopontin, rheumatoid arthritis, 23C3, epitope, antibody structure

## Abstract

Osteopontin plays an important role in the development and perpetuation of rheumatoid arthritis (RA). Antibodies targeting osteopontin have shown promising therapeutic benefits against this disease. We have previously reported a novel anti-RA monoclonal antibody, namely, 23C3, and shown it capable of alleviating the symptoms of RA in a murine collagen-induced arthritis model, restoring the cytokine production profile in joint tissues, and reducing T-cell recall responses to collagen type II. We describe here the crystal structure of 23C3 in complex with its epitope peptide. Analyses of the complex structure reveal the molecular mechanism of osteopontin recognition by 23C3. The peptide folds into two tandem β-turns, and two key residues of the peptide are identified to be critical for the recognition by 23C3: TrpP43 is deeply embedded into a hydrophobic pocket formed by AlaL34, TyrL36, LeuL46, TyrL49, PheL91, and MetH102 and therefore has extensive hydrophobic interactions with 23C3, while AspP47 has a network of hydrophilic interactions with residues ArgH50, ArgH52, SerH53, and AsnH56 of the antibody. Besides the complementarity-determining region loops, the framework region L2 of 23C3 is also shown to interact with the epitope peptide, which is not common in the antibody–antigen interactions and thus could be exploited in the engineering of 23C3. These results not only provide valuable information for further improvement of 23C3 such as chimerization or humanization for its therapeutic application, but also reveal the features of this specific epitope of osteopontin that may be useful for the development of new antibody drugs against RA.

The pathogenesis of rheumatoid arthritis (RA), a chronic autoimmune disease, involves accumulation of T lymphocytes in the synovial compartment and destruction of cartilage and bone.^1–3^ Several antirheumatic drugs are available in the market including two monoclonal antibody (mAb) drugs against tumor necrosis factor-α, namely, Infliximab (Centocor) and Adalimumab (Abbott). However, about 20–30% of the patients treated do not respond to the antibodies and some drugs lose efficacy or accumulate toxicity over time, demanding development of new strategies for the treatment of RA, probably against other factors involved in the pathogenesis of RA.^4–6^

One such target is osteopontin, also known as early T-cell activation gene-1. It is one of the major noncollagenous bone matrix proteins and is expressed at elevated level at the inflammation site in various cell types including epithelial cells, mesenchymal cells, and immune cells.^7^ The protein is usually phosphorylated and can be cleaved by thrombin *in vivo*. When cleaved, the N-terminal fragment is capable of binding to various cell surface receptors such as integrins α_v_β_3_, α_v_β_1_, α_v_β_5_, and α_8_β_1_ via its RGD motif,^8–10^ and α_9_ and α_4_ integrins via a crypt region (162SVVYGLR168 of human osteopontin);^11–13^ both the N-terminal and C-terminal fragments can bind to CD44 variants with the assistance of β_1_ integrins independently of the RGD sequence.^14^ Considering the various interactions between osteopontin and multiple cell receptors, it is not surprising that osteopontin exerts diverse functions in immunity, infection, and cancer (reviewed in the work of Denhardt and Guo^15^). Specifically, the multifaceted protein is involved in various aspects of immunity: osteopontin is considered an early component of cell-mediated immunity^16^ and seems to play a complicated role in T_H_2 effector responses^17^ and to mediate natural killer T-cell function.^18^ Moreover, osteopontin prevents monocyte apoptosis^19^ and seems to regulate the expression of cell-surface CD44 on macrophages and to modulate CD44 activities.^20^

An important role of osteopontin in amplification and perpetuation of RA is suggested by the confined expression of osteopontin in synovial fluid (SF) and synovial tissue of RA patients^21,22^ and the co-expression of osteopontin receptors (α_v_, β_1_ and CD44) in T cells in SF.^21^ In addition, when T cells are treated with RA-SF, osteopontin expression is elevated; on the other hand, overexpression of osteopontin induces proinflammatory chemokines and cytokines and activates nuclear factor-kappa B.^21^ Osteopontin deficiency is consistently shown to prevent the destruction of joint cartilage and joint swelling through suppression of chondrocyte apoptosis and angiogenesis in the RA model.^21^

Due to the association between osteopontin and RA, several anti-osteopontin antibodies have been developed and studies of these antibodies in animal RA models show promising results.^23–25^ The antibody M5 recognizes and binds to a cryptic epitope of thrombin-cleaved mouse osteopontin (147SLAYGLR153) and thereby blocks the binding of cleaved osteopontin to α_9_ and α_4_ integrins, which result in abrogation of monocyte migration.^18^ Treatment with M5 leads to inhibition of synovial proliferation, bone resorption, and inflammatory reaction in arthritic joints in the murine RA model induced by mAb/lipopolysaccharides.^24^ Another mAb, 2K1 or its chimeric form C2K1, recognizes the equivalent epitope region of human osteopontin (162SVVYGLR168) and is shown to ameliorate collagen-induced arthritis (CIA) in cynomolgus monkey.^23^

Recently, we have developed a novel murine anti-osteopontin mAb, namely, 23C3, which is of great potential in treatment of RA.^25^ Administration of 23C3 to mice with CIA not only strongly suppresses the development of CIA but also decreases the severity of the existing arthritis. Mice treated with 23C3 showed a cytokine production profile in joint tissues similar to that of the wild-type mice and reduced T-cell recall responses to collagen type II. We have also produced another murine mAb, F8E11, which is also included for the same assay, however, it shows much weaker efficacy for the treatment of the established disease in the murine model.^25^ As both F8E11 and 23C3 are capable of inhibiting monocyte migration, the robust therapeutic effect of 23C3 may be attributed to its ability to promote the apoptosis of activated T cells by inhibition of nuclear factor-kappa B and alteration of the expression of preapoptotic proteins,^25^ which may be unique, as neither M5 nor 2K1 is reported to be capable of induction of cell apoptosis. Intriguingly, 23C3 recognizes a distinct epitope motif (43WLNPDP48) of the full-length osteopontin, which may account for the differences.^25^ In this study, we report the crystal structure of the Fab fragment of 23C3 in complex with its epitope peptide of osteopontin. Our results reveal the structural basis for the 23C3 recognition of osteopontin and provide hints for further development of mAb drugs for the treatment of RA.

## The structure of the 23C3 Fab

**Fig. 1:**
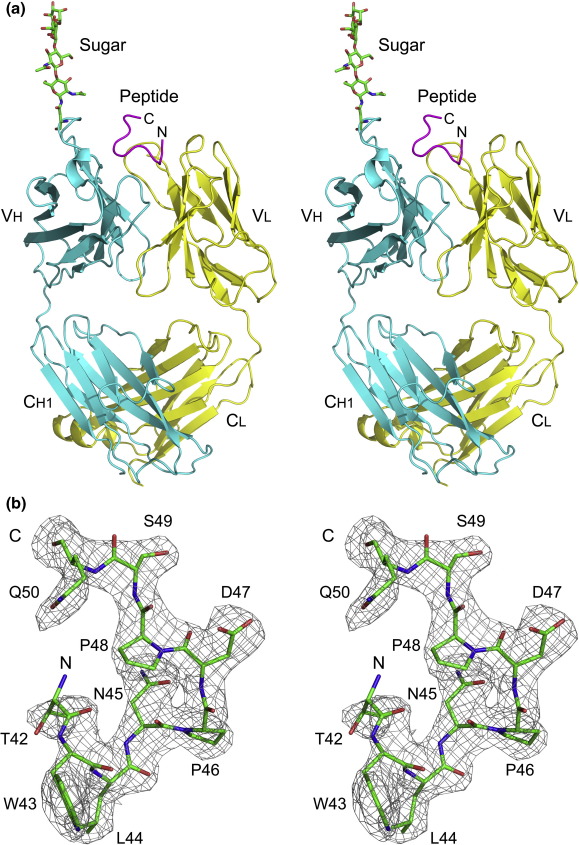
Overall structure of the 23C3 Fab–epitope peptide complex. (a) Stereoview of the overall structure of the 23C3 Fab–epitope peptide complex. The 23C3 Fab light chain is colored yellow, the heavy chain is cyan, and the bound peptide is purple. The sugar chain is shown with a ball-and-stick model. (b) A stereo view representation of SIGMAA-weighted 2*F*_o_ − *F*_c_ map (1σ contour level) for the bound epitope peptide. The peptide is shown with a ball-and-stick model.

The Fab fragment of the anti-osteopontin mAb 23C3 was co-crystallized with a 12-mer peptide (40VATWLNPDPSQK51) that contains the previously mapped epitope region of osteopontin recognized by 23C3.^25^ The structure of 23C3 Fab in complex with the epitope peptide was determined to 2.8 Å resolution with an *R*−factor of 24.8% and a free *R*−factor of 29.2% (Table 1, Fig. 1). There is one Fab–peptide complex in an asymmetric unit. Overall, the complex exhibits good geometry and has well-defined electron density even at the conserved loop region between GlyH131 and SerH137, which is disordered in many other Fab structures.

The Fab fragment displays a canonical Fab structure with four immunoglobulin domains of V_L_, C_L_, V_H_, and C_H1_ (Fig. 1a). The elbow angle relating the disposition of V_H_ to V_L_ and C_H1_ to C_L_ is about 150°, consistent with the fact that 23C3 has a κ light chain.^26^ The complementarity-determining region (CDR) loops L1, L2, L3, H1, and H2 belong to Chothia canonical classes 2, 1, 1, 1, and 4, respectively.^27^ The CDR loop H3 of 23C3 contains only five residues, which is significantly shorter than the average length of 8.7 residues for murine mAbs.^28^ Interestingly, the Fab is glycosylated at AsnH57 of CDR H2 (Fig. 1a). An N-linked core tetrasaccharide (MANα-1,3MANβ-1,4GlcNAcβ-1,4GlcNAcβ-1-AsnH57) was modeled without ambiguity. N-linked glycosylation of mAbs in both framework regions (FWRs)^29^ and CDR loops^30,31^ has been reported, and sugar chains on the CDR loops may also participate in the interactions with antigens.^31^ In our structure, there is no interaction between the sugar chain and the bound peptide. Due to the lack of a structure of the full-length osteopontin, the possibility that the sugar chain might be involved in the recognition of the full-length antigen cannot be excluded.

## The structure of the bound peptide

The bound peptide has well-defined electron density (from ThrP42 to GlnP50) except for several terminal residues (Fig. 1b). As shown in Fig. 1, the peptide displays a twisted structure and is composed of the N-terminal two tandem β-turns (β-turn is the most common structural element of antigens in mAb–peptide complexes^32^) and the two C-terminal extended residues (SerP49 and GlnP50). The first β-turn (ThrP42 to AsnP45) is of type I and the second (AsnP45 to ProP48) is of type VIII. The conformation of the peptide is mainly stabilized by the interactions between the peptide and the Fab (see the discussion later), while a hydrogen bond between the main-chain amide of AspP47 and the side-chain carbonyl of AsnP45 (2.88 Å) also contributes to the stability.

## The interactions between the Fab and peptide

**Fig. 2:**
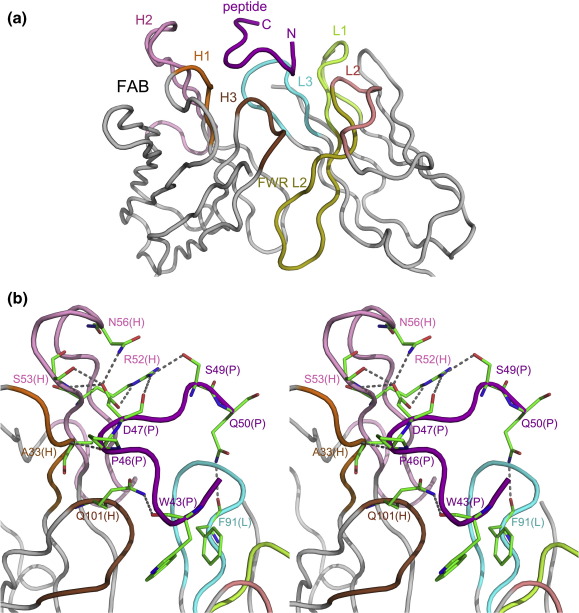

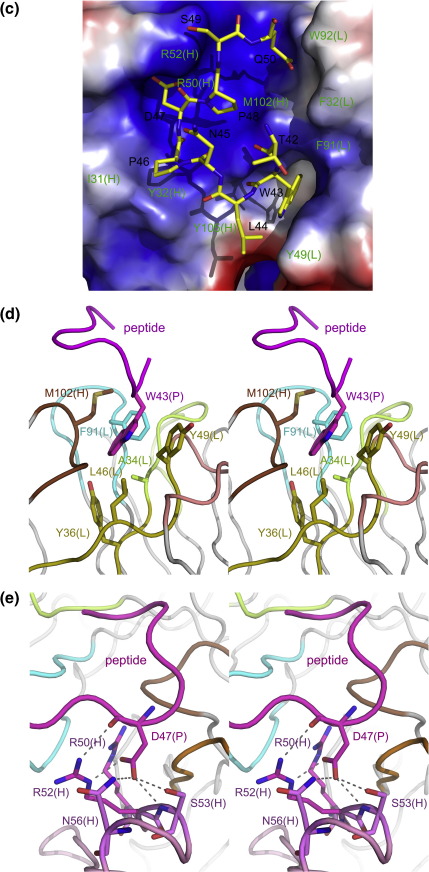
The interactions between the 23C3 Fab and the bound peptide. (a) An enlarged view of the interface surface. The peptide is bound at a pocket formed mainly by CDRs and interacts with CDRs L1, L3, H1, H2, and H3 and FWR L2. The Fab CDR L1 is colored lemon; L2, salmon; L3, cyan; H1, orange; H2, pink; H3, brown; FWR L2, olive; and other FWRs, silver. The peptide is colored purple. (b) A stereo view showing the hydrogen bonds between residues of the 23C3 Fab and residues of the epitope peptide. The color coding is the same as above. The residues that participate in the hydrogen-bonding interactions are shown with ball-and-stick models. (c) An electrostatic potential surface of the 23C3 Fab at the interface. The Fab accommodates the peptide with great structural and chemical complementarity. (d) The extensive hydrophobic interactions between the Fab and the key residue TrpP43 of the epitope peptide. Residues AlaL34 of CDR L1, PheL91 of CDR L3, MetH102 of CDR H3, and TyrL36, LeuL46, and TyrL49 of FWR L2 form a deep hydrophobic pocket to accommodate TrpP43 of the peptide. (e) The hydrophilic recognition of AspP47 by the Fab. AspP47 makes hydrogen-bonding interactions with SerH53, AsnH56, and ArgH52. It also forms electrostatic interactions with residues ArgH50 and ArgH52 of CDR H2. The color coding is the same as in (a).

In the complex structure, the peptide is bound in a pocket formed by the CDR loops and the tip of the FWR L2 (defined by Kabat definition). CDR loops L1, L3, H1, H2, and H3 participate in the interactions with the peptide, while loop L2 makes no direct contact with the peptide, as frequently observed in antipeptide or antihapten Fab structures (Fig. 2a).^33^ Uncommonly, some residues of the FWR L2 (including TyrL36, LeuL46, and TyrL49) have van der Waals contacts with the peptide, probably due to the deep embedment of the side chain of TrpP43 into the Fab (see detailed discussion later). The buried surface area (BSA) of the interaction interface is about 591.3 Å^2^ within the average range of 400–700 Å^2^ for mAb–peptide complexes,^34^ accounting for 47.4% of the total peptide surface area. The shape complementarity value of this complex is 0.80, much higher than the average value (0.64–0.68) for antibody–antigen complexes.^35^ Between the Fab and the peptide, nine hydrogen bonds, one salt bridge, and 126 van der Waals contacts are formed (Tables 2 and S1 and Fig. 2b and c). The CDR loops L1, L3, H1, H2, and H3 contribute 23.0 Å^2^ (5.0%), 74.2 Å^2^ (15.5%), 76.1 Å^2^ (15.9%), 98.6 Å^2^ (20.6%), and 132.9 Å^2^ (27.7%) of the BSA, respectively. Additionally, FWR L2 also has 74.4 Å^2^ (15.5%) of the BSA. These results are consistent with the notion that the H chain makes more interactions than the L chain when a mAb binds to a small-molecule antigen or a peptide antigen.^32^

Analysis of the complex structure shows that all of the detected nine residues of the peptide interact with the 23C3 Fab and, particularly TrpP43 and AspP47, are the key residues recognized by 23C3. Specifically, the aromatic residue TrpP43 has a large hydrophobic indole group buried in a deep hydrophobic cage formed by AlaL34, TyrL36, LeuL46, TyrL49, PheL91, and MetH102, forming 46 van der Waals contacts and contributing a large BSA of 233.0 Å^2^ (Fig. 2d). This residue alone contributes 36.5% of all the van der Waals contacts between the Fab and the peptide and 33.1% of all the BSA of the peptide. Additionally, the main-chain carbonyl of TrpP43 forms a hydrogen bond with the side-chain amide of GlnH101 of CDR loop H3. The acidic residue AspP47 is embedded into a positively charged cleft to form electrostatic interactions with ArgH50 and ArgH52 (Fig. 2e). The side chain of AspP47 has three hydrogen-bonding interactions with SerH53 and AsnH56, and its main-chain carbonyl forms a hydrogen bond with the side chain of ArgH52 as well. These interactions make AspP47 the most important contributor for the hydrophilic interactions between the peptide and the Fab.

To further validate the critical roles of TrpP43 and AspP47 in the binding of 23C3, we carried out structural studies of the 23C3 Fab in complexes with the mutant peptides containing single mutation W43A or D47A, or double mutations W43A/D47A. The crystal structure of the 23C3 Fab in complex with the W43A mutant peptide was determined at 2.8 Å resolution with an *R*−factor of 24.1% and a free *R*−factor of 29.7% (Table 1). The structure of the mutant complex resembles that of the wild-type peptide complex (Fig. S1). Superposition of the two complexes yields an RMSD of 0.4 Å for 436 aligned C^α^ atoms. In this mutant peptide complex, only seven (LeuP44 to GlnP50) of the nine peptide residues (ThrP42 to GlnP50) observed in the wild-type peptide complex could be defined with clear electron density and the two N-terminal residues ThrP42 and AlaP43 are disordered, indicating that the binding of this region with the Fab is substantially impaired due to the loss of the hydrophobic interactions caused by the W43A mutation. Moreover, crystallization of the 23C3 Fab in complex with the D47A or W43A/D47A mutant peptide was unsuccessful so far. It is most likely that the D47A mutation of the peptide would disrupt the extensive hydrophilic interactions of the peptide with the Fab and lead to the loss of the peptide's ability to bind the Fab. These results support our notion that TrpP43 and AspP47 are essential for the binding of 23C3.

Although less important, the other residues within the mapped epitope 43WLNPDP48^25^ are also involved in the binding of the Fab (Fig. 2b, Tables 2 and S1). The side chains of residues LeuP44 and AsnP45 stretch out against the Fab–peptide interaction interface and each forms 3 van der Waals contacts. The main-chain carbonyl of ProP46 forms a hydrogen bond with the main-chain amide of AlaH33. The side chain of ProP46 is surrounded by a hydrophobic concave formed by residues LeuH31 and TyrH32 and forms 14 van der Waals contacts. The side chain of ProP48 is covered by AsnP45 and points to the interface forming 7 van der Waals contacts. Besides the 43WLNPDP48 motif, the other three residues (ThrP42, SerP49, and GlnP50) also contribute some van der Waals contacts with the Fab. The side chains of SerP49 and GlnP50 each form a hydrogen bond with the side chain of ArgH52 and the main-chain carbonyl of PheL91, respectively.

It is worth noting that besides CDRs, three conserved κ chain residues of FWR L2 (TyrL36, LeuL46, and TyrL49) are also involved in the interactions between the peptide and the Fab in addition to their typical roles in the interactions between V_L_ and V_H_ domains. In our structure, they all make van der Waals contacts with the peptide due to the deep embedment of TrpP43, which is rare, as residues TyrL36 and LeuL46 generally do not participate in the Fab–antigen interactions and TyrL49 is only occasionally found in interactions between the Fab and nuclear acids or hapten antigens.

In summary, 23C3 is a novel anti-osteopontin mAb with great potential for the treatment of RA. By determination of the crystal structure of 23C3 Fab in complex with its epitope peptide, we have revealed the interactions between the antibody and the peptide and identified the most important determinants for this epitope (TrpP43 and AspP47). We have also showed that FWR L2 plays an important role in the recognition and binding of the epitope by the antibody, suggesting that this region should be exploited in the development of chimeric or humanized antibodies. Besides, the detailed conformation of the epitope peptide may also be useful for the design of peptide vaccines against RA. As an example, a chemically constrained peptide mimicking the structural conformation of the epitope peptide of gp41 shows much stronger binding with the neutralizing HIV antibody 4E10 than the free peptide and therefore would be a better immunogen.^36^

## Protein Data Bank accession code

The coordinates and structural factors of the 23C3 Fab fragment in complexes with its epitope peptide and the W43A mutant peptide have been deposited in the RCSB Protein Data Bank with accession codes 3CXD and 3DSF, respectively.

## Figures and Tables

**Table 1:** Summary of diffraction data and structure refinement statistics

*Diffraction data*		
Crystal	23C3-epitope peptide	23C3-W43A peptide
Wavelength (Å)	1.5418	1.5418
Space group	*P*2_1_2_1_2_1_	*P*2_1_2_1_2_1_
Cell parameters		
*a*/*b*/*c* (Å)	42.3/92.0/122.5	41.7/92.1/122.1
Resolution range (Å)	20.0–2.80 (2.90–2.80)^a^	20.0–2.80 (2.90–2.80)
Observed reflections	82,967 (8415)	66,404 (6296)
Unique reflections [*I*/σ(*I*) > 0]	12,316 (1192)	12,155 (1171)
Average redundancy	6.7 (7.1)	5.4 (5.3)
Average *I*/σ(*I*)	5.0 (2.1)	6.3 (3.1)
Completeness (%)	98.4 (98.4)	99.4 (99.4)
Wilson *B*-factor (Å^2^)	58.9	55.6
*R*_merge_ (%)^b^	13.5 (29.3)	8.4 (22.9)

*Statistics of refinement and model*		
No. of reflections [*F*_o_ > 0σ(*F*_o_)]		
Working set	11,470 (836)	11,476 (824)
Free *R* set	613 (32)	609 (35)
*R*−factor (%)^c^	24.8 (37.3)	24.1 (31.5)
Free *R*−factor (%)	29.2 (47.7)	29.7 (39.5)
No. of protein residues	438	436
Fab/Peptide	429/9	429/7
No. of sugar residues	4	4
Average *B*-factor of all atoms (Å^2^)	35.4	33.5
Fab/Peptide	34.8/40.7	33.0/33.2
Sugar	64.2	62.1
r.m.s.d. bond lengths (Å)	0.006	0.010
r.m.s.d. bond angles (°)	1.003	1.171
Luzzati atomic positional error (Å)	0.46	0.42
Ramachandran plot (%)		
Most favored regions	84.9	86.8
Allowed regions	14.0	11.4
Generously allowed regions	0.3	1.1
Disallowed regions	0.8	0.8

a Numbers in parentheses refer to the highest-resolution shell.

b *R*_merge_ = ∑_*hkl*_∑_*i*_|*I*_*i*_(*hkl*)_*i*_ − 〈*I*(*hkl*)〉|/∑_*hkl*_∑_*i*_*I*_*i*_(*hkl*).

c *R*−factor = ∑_*hkl*_ ||*F*_o_|−|*F*_c_||/∑_*hkl*_ |*F*_o_|.

**Table 2:** The hydrophilic interactions between 23C3 Fab and the peptide

Peptide atoms	Fab atoms	CDR loops	Distance (Å)
TrpP43-O	GlnH101-N^ε2^	H3	2.9
ProP46-O	AlaH33-N	H1	3.0
AspP47-O^δ1^	SerH53-N	H2	3.0
AspP47-O^δ1^	SerH53-O^γ^	H2	2.8
AspP47-O^δ1^	AsnH56-N^δ2^	H2	2.9
AspP47-O^δ2^	ArgH52-N^ε^	H2	2.7
AspP47-O	ArgH52-N^η1^	H2	3.1
SerP49-O^γ^	ArgH52-N^η1^	H2	3.0
GlnP50-N^ε2^	PheL91-O	L3	3.0
