# Supplementary material for: Molecular Basis of Recognition of Human Osteopontin by 23C3, a Potential Therapeutic Antibody for Treatment of Rheumatoid Arthritis
Source: J Mol Biol. 2008 Oct 17;382(4):835–42. doi: 10.1016/j.jmb.2008.07.075 (PMC2793339; doi:10.1016/j.jmb.2008.07.075)
Supplement: Figure S1 [file mmc1.doc]

**Supplementary Materials and Methods**

**Protein preparation and purification**

The mAb 23C3 against human osteopontin was produced by hybridoma cells.^1^ The purified antibody was digested by papain and the Fab fragment of 23C3 was purified as described previously.^2^ The purity of the Fab fragment was confirmed by SDS PAGE analysis. The 12-mer peptide (VATWLNPDPSQK) corresponding to residues 40-51 of human osteopontin and three mutant peptides (W43A, D47A, and W43A/D47A) were all synthesized at Shanghai HD Bioscience Company.

**Crystallization and data collection**

The purified 23C3 Fab fragment was dialyzed into a stock buffer (100 mM NaCl, 10 mM Tris-HCl, pH 8.0) and concentrated to 15 mg/ml. The Fab-peptide complex was prepared by mixing the Fab and the peptide with a molar ratio of 1:5 at 4 °C for 12 hours, and then set for crystallization screening with hanging drop vapor diffusion method at 4 °C. In a drop containing 0.7 μl of the Fab-peptide mixture and 0.7 μl of the reservoir solution (0.2 M di-sodium tartrate and 20% PEG3350) equilibrated against 400 μl of the reservoir solution, square shaped crystal of the 23C3 Fab in complex with its epitope peptide grew to a final dimension of 0.2 × 0.2 × 0.1 mm^3^ after 15 days. The crystal of the 23C3 Fab in complex with the W43A mutant peptide grew in the condition of 0.2 M di-ammonium hydrogen phosphate and 20% PEG3350. The trials to crystallize the 23C3 Fab alone or in complex with the D47A or W43A/D47A double mutant peptide were unsuccessful so far. The crystals used for diffraction data collection was cryo-protected in the reservoir solution supplemented with 20% PEG400 and then flash cooled to -180 °C. Diffraction data were collected with an in-house Rigaku R-AXIS IV++ diffractometer and processed with the program CrystalClear (Rigaku). The statistics of the diffraction data are summarized in Table 1.

**Structure determination, refinement and analysis**

The structure of 23C3 Fab in complex with its epitope peptide of osteopontin was determined by the molecular replacement method using the program Phaser^3^ with an anti-Estradiol Fab fragment^4^ (PDB entry 1JHK) as the search model. After several rounds of refinement in program Refmac5^5^ and manual model building with the program Coot^6^, the electron density is sufficiently clear for tracing the peptide and the sugar without ambiguity. All the data were used for refinement except 5% randomly chosen reflections were set aside for free R factor cross-validation throughout the refinement. The structure of the 23C3 Fab in complex with the W43A mutant peptide was determined and refined using the same protocol as the structure of the 23C3 Fab in complex with the wild-type peptide. The geometry of the final models were analyzed with the program Procheck.^7^ The summerized refinement statistics are listed in Table 1. The shape complementarity value Sc was determined by the program Sc2.0^8^ with default parameters. Buried surface areas were calculated using the program Areaimol^9^ with a probe radius of 1.4 Å. Hydrogen bonds and van der Waals contacts were calculated by using the program Contact^10^ followed by manual check of proper geometry. The elbow angle was determined with web based program Rbow.^11^ All the graphics were generated by using the program Pymol ([http://www.pymol.org](http://www.pymol.org/)).

**Reference**

1. Fan, K., Dai, J., Wang, H., Wei, H., Cao, Z., Hou, S., Qian, W., Wang, H., Li, B., Zhao, J., Xu, H., Yang, C. & Guo, Y. (2008). Treatment of collagen-induced arthritis with an anti-osteopontin monoclonal antibody through promotion of apoptosis of both murine and human activated T cells. *Arthritis Rheum.* **58**, 2041-2052.

2. Du, J., Wang, H., Zhong, C., Peng, B., Zhang, M., Li, B., Hou, S., Guo, Y. & Ding, J. (2008). Crystal structure of chimeric antibody C2H7 Fab in complex with a CD20 peptide. *Mol. Immunol.* **45**, 2861-2868.

3. McCoy, A. J., Grosse-Kunstleve, R. W., Storoni, L. C. & Read, R. J. (2005). Likelihood-enhanced fast translation functions. *Acta Crystallogr.* **D61**, 458-64.

4. Lamminmaki, U. & Kankare, J. A. (2001). Crystal structure of a recombinant anti-estradiol Fab fragment in complex with 17β-estradiol. *J. Biol. Chem.* **276**, 36687-94.

5. Murshudov, G. N., Vagin, A. A. & Dodson, E. J. (1997). Refinement of macromolecular structures by the maximum-likelihood method. *Acta Crystallogr.* **D53**, 240-55.

6. Emsley, P. & Cowtan, K. (2004). Coot: model-building tools for molecular graphics. *Acta Crystallogr.* **D60**, 2126-32.

7. Laskowski, R. A., MacArthur, M. W., Moss, D. S. & Thornton, J. M. (1993). PROCHECK: a program to check the stereochemical quality of protein structures. *J. Appl. Cryst.* **26**, 283-91.

8. Lawrence, M. C. & Colman, P. M. (1993). Shape complementarity at protein/protein interfaces. *J. Mol. Biol.* **234**, 946-50.

9. Lee, B. & Richards, F. M. (1971). The interpretation of protein structures: estimation of static accessibility. *J. Mol. Biol.* **55**, 379-400.

10. Collaborative Computational Project Number 4. (1994). The CCP4 suite: programs for protein crystallography. *Acta Crystallogr.* **D50**, 760-3.

11. Stanfield, R. L., Zemla, A., Wilson, I. A. & Rupp, B. (2006). Antibody elbow angles are influenced by their light chain class. *J. Mol. Biol.* **357**, 1566-74.

**Supplementary Figure Legend**

**Figure S1.** Structural comparison between the crystal structures of the 23C3 Fab in complex with the wild-type epitope peptide (green) and the D43A mutant peptide (yellow). The structural comparison shows that when Trp^P43^ is mutated, the 23C3 Fab (wheat) adopts a similar conformation as that of the 23C3 Fab (grey) in complex with the wild-type peptide. The conformations of the peptides are similar except that the two N-terminal residues of the W43A mutant peptide are disordered. Several residues on both peptides are shown with ball-and-stick models and labeled for reference.

**Table S1. The buried surface area (BSA) of each peptide residue and the van der Waals Contacts between the peptide residues and the interacting CDR residues.**

**Peptide residues BSA (Å^2^) Contacting Fab residues (≤ 4 Å)**

Thr^P42^ (3) ^a^ 23.1 Gln^H101^ (1), Met^H102^ (2)

Trp^P43^ (46) 233.0 Ala^L34^ (2), Tyr^L36^ (1), Leu^L46^ (5), Tyr^L49^ (12), Phe^L91^ (6), Gln^H101^ (2), Met^H102^ (8), Gly^H103^ (5), Asp^H104^ (5)

Leu^P44^ (3) 40.9 Tyr^L49^ (3)

Asn^P45^ (3) 5.3 Gln^H101^ (3)

Pro^P46^ (21) 104.3 Ile^H31^ (5), Tyr^H32^ (9), Ala^H33^ (3), Gln^H101^ (4)

Asp^P47^ (28) 114.6 Ile^H31^ (1), Ala^H33^ (2), Arg^H52^ (16), Ser^H53^ (5), Asn^H56^ (4)

Pro^P48^ (7) 40.8 Arg^H52^ (3), Gln^H101^ (3), Met^H102^ (1)

Ser^P49^ (7) 34.1 Arg^H52^ (6), Asn^H56^ (1)

Gln^P50^ (8) 107.2 Phe^L91^ (2), Trp^L92^ (5), Gly^L93^ (1)

^a^ Numbers in parentheses refer to the number of van der Waals contacts.

**Supplementary Figure S1**

**
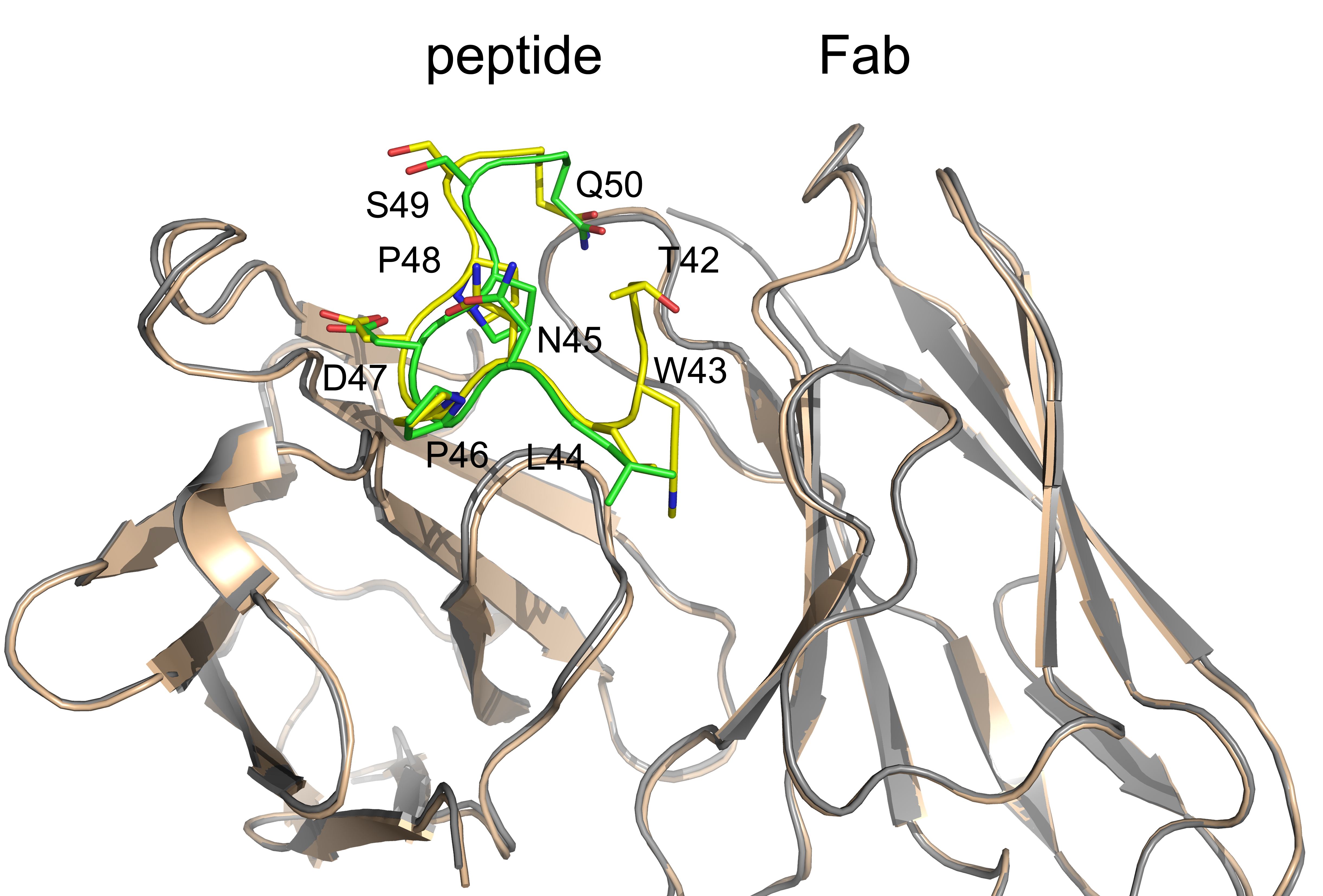
**
